# Letter to Editor: Development of transdisciplinary approach scale for healthcare professionals

**DOI:** 10.1002/pcn5.70391

**Published:** 2026-08-07

**Authors:** Muhammad Shehryar

**Affiliations:** ^1^ Bachelor of Medicine and Bachelor of Surgery Sahiwal Medical College Sahiwal Punjab Pakistan

To the Editor,

I read with great interest the recently published article, “Development of transdisciplinary approach scale for healthcare professionals”^1^ by Watanabe et al. The authors' contribution in developing the first validated instrument specifically designed to measure transdisciplinary‐specific competencies, like role release and shared conceptual frameworks among mental health professionals, is important and timely. A systematic scale development and the diversity of 14 professional groups and the responsiveness to educational intervention are valuable contributions to the field. However, a few limitations should be discussed.

There appear to be discrepancies between the numbers of males and females for the various subgroups in Table 2 of the original article by Watanabe et al., for which the subsample sizes do not seem to match the reported totals for the various professions. Clarification, and where necessary formal correction, of these figures would strengthen confidence in the descriptive data reported and help readers accurately interpret the composition of the study sample.

In addition to this point, a few methodological considerations are worthy of brief mention, although most have been justly mentioned by the authors as limitations in the original paper. The two‐factor structure of Transdisciplinary Approach Scale (TDAS) was obtained using exploratory factor analysis with a subject‐to‐item ratio of approximately 6:1, which is below the ratio generally thought to be necessary for the factor to be stable and replicable, namely 10:1.^2^ It is difficult to rule out alternative explanations such as Hawthorne effect or natural professional maturation because the pre‐post improvements after participating in PsySEPTA (Cohen's *d* = 0.64–0.73) have not been compared with a control group.^3^ The participants were also self‐selected into a program that was explicitly transdisciplinary, which could lead to selection bias raising baseline scores and observed gains compared to a more representative clinical sample.^4^ Finally, the measurement invariance over the 12 months follow‐up period was not formally tested and would be useful to ensure that the observed changes in scores were due to actual improvement in the measured construct and not to participants' different interpretations of the items over time.^5^ As the authors themselves acknowledge these constraints and call for further validation, these points are noted here for completeness rather than as unresolved concerns requiring further response.

To summarize, the TDAS is a commendable first attempt to establish transdisciplinary collaboration as a measurable phenomenon in psychiatric practice. The most pressing issue for the authors to address is the apparent numerical discrepancy in Table 2, which warrants formal clarification or correction. The other methodological considerations mentioned above, which the authors are already aware of and which were mentioned above, will be useful to consider in the future extensive validation studies that the authors call for.

## DECLARATION OF GENERATIVE AI AND AI‐ASSISTED TECHNOLOGIES IN THE WRITING PROCESS

After the preparation of this Letter, the author used Anthropic's Claude Sonnet 4.6 in order to enhance the readability and language of the manuscript. After using this tool, the author reviewed and edited the content as needed and takes full responsibility for the content of the publication.

## AUTHOR CONTRIBUTIONS

Muhammad Shehryar is the sole author of this letter and is responsible for its conception, drafting, and final approval.

## CONFLICT OF INTEREST STATEMENT

The author declares no conflicts of interest.

## ETHICS APPROVAL STATEMENT

N/A.

## PATIENT CONSENT STATEMENT

N/A.

## CLINICAL TRIAL REGISTRATION

N/A.

## Data Availability

Data sharing not applicable to this article as no datasets were generated or analyzed during the current study.
